# Innovative teaching methods for capacity building in knowledge translation

**DOI:** 10.1186/1472-6920-11-85

**Published:** 2011-10-14

**Authors:** Hayfaa A Wahabi, Lubna A Al-Ansary

**Affiliations:** 1 Sheikh Bahmdan Chair for Evidence-Based Healthcare and Knowledge Translation, King Saud University, College of Medicine, Riyadh, Kingdom of Saudi Arabia

## Abstract

**Background:**

In some current healthcare settings, there is a noticeable absence of national institutions committed to the synthesis and use of evidence in healthcare decision- and policy-making. This absence creates a need to broaden the responsibilities of healthcare providers to include knowledge brokering and advocacy in order to optimize knowledge translation to other stakeholders, especially policy-makers. However, this process requires practitioners and researchers to acquire certain types of knowledge and skills. This article introduces two innovative methods for capacity building in knowledge translation (KT).

**Methods:**

During a workshop aimed at preparing 21 trainers in evidence-based medicine, two innovative methods were used: (1) debate and (2) a knowledge translation project (KTP). The main objective of the debates approach was to strengthen participants' critical thinking abilities by requiring them to search for and appraise evidence and defend their arguments. The KTP was used to introduce participants to the essential steps of knowledge translation and to suggest an extended role for healthcare practitioners, i.e., using evidence to manage not only individual patients but also to a community of patients. Participants' performances were assessed according to a pre-designed scheme. At the end of the workshop, participants' opinions and experiences with the innovative teaching methods were evaluated based on their answers to a questionnaire and the results of small-group discussions.

**Results:**

The participants performed well in both the debate and KTP methods. During post-workshop group discussions, they indicated that the debate approach had added a new dimension to their evidence-based medicine skills by adding purpose and motivation. However, they felt that their performances would have been better if they had been offered practical demonstrations of how to conduct the debate. The participants indicated that the KTP enhanced their understanding of the relationships between evidence and implementation, and motivated them to investigate public health problems in addition to individual patient problems. However, some participants maintained that these issues fell outside the scope of their role as doctors.

**Conclusion:**

Debates and evidence implementation through KTP are generally well accepted by healthcare practitioners as methods by which they can improve their skills in KT.

## Background

The knowledge-practice gap is an internationally recognized obstacle in translating evidence into practice [1].

Family medicine doctors constitute the main source of manpower for 2037 primary healthcare centers all over the Kingdom of Saudi Arabia, besides the primary healthcare units in the University Hospitals. In addition to their clinical duties, family doctors are charged with administrative and managerial duties, especially in rural areas.

The Saudi Ministry of Health (MOH), in cooperation with academic institutions such as King Saud University (KSU), conducts various training programs for family medicine physicians. Most of these programs are aimed at continuing professional development. However, in 2009, a new diploma program was introduced, in which the Saudi Commission of Health Specialties (SCHS) created a specialty in family medicine. As part of this effort, the MOH introduced a structured training program to certify family medicine consultants as trainers in the diploma program. The prospective trainers were selected from a group of applicants who had completed their residency program and were board-certified family medicine physicians. Priority was given to applicants with previous experience in teaching residents.

The Chair of Evidence-Based Healthcare and Knowledge Translation (CEBHC-KT) was established in 2008 as part of a research promotion program launched by KSU in Riyadh.

Since 1997, the Family and Community Medicine Department of KSU has run regular workshops for healthcare professionals from the MOH and from other governmental and private institutions. These workshops address EBM and critical appraisal of the literature. Following the establishment of the CEBHCKT, the task of training in EBM was taken over by the Chair and became part of its activities. In 2009, the Saudi MOH commissioned the CEBHC-KT to deliver a module on advanced evidence-based healthcare as part of the Train the Trainers course. The course is held annually and is expected to continue providing trainers charged with the training of family medicine residents and specialists at the 10 centers recognized by the SCHS.

Evidence-based medicine (EBM) was introduced in Saudi Arabia over a decade ago and the concept was soon endorsed by many academic and service institutions, which then sought critical appraisal for this approach in workshops aimed at teaching its basic concepts [2]. This activity contributed to the training of a group of facilitators with knowledge and skills in evaluating evidence and in communicating that knowledge to clinicians and other healthcare workers. Indeed, these trainers played a pioneering role in spreading the concept of EBM among healthcare providers. However, like many other countries in the Middle East, in Saudi Arabia there is a noticeable absence of national institutions for the synthesis and use of evidence in healthcare decision-making, due to a lack of communication channels between health policy makers and EBM experts.

This article reports the efforts of the CEBHC-KT to advance training in EBM in Saudi Arabia. The goal was to go beyond critical appraisal of the biomedical literature by building skills in knowledge brokering and by establishing networks between EBM experts and the end-users of that evidence, especially but not limited to policy makers.

## Methods

### Participants

Twenty-one family medicine consultants (7 women, 14 men, age range 28-53 years) were selected by the MOH from different regions of Saudi Arabia and invited to participate in the second 'Train the Trainers workshop' in order to enhance their abilities as clinical tutors and trainers for the Family Medicine diploma program.

### Pre-workshop assessment and objectives of the workshop

Knowledge of the aspects of EBM listed below was a prerequisite for participating in the innovative learning/teaching sessions:

1. The five steps of EBM (formulating clinical question, searching for evidence, appraising the evidence, applying the evidence, and evaluating the outcome of implementation). Knowing how to search the main databases such as Medline and the Cochrane Library. Demonstrable skills in critical appraisal of the literature, especially regarding clinical trials and interventional studies.

2. Knowledge of the hierarchy of evidence and the importance of implementing high-level evidence in practice.

3. Skills and knowledge in the quantification and interpretation of effect size of the intervention according to values such as the number needed to treat (NNT) and relative risk reduction (RRR).

To ensure that the participants had the required knowledge and skills in EBM, two weeks before the workshop began, applicants took a formative assessment test (the Fresno test) [3], and completed a questionnaire about their practices and attitudes towards evidence-based healthcare. A satisfactory result on the Fresno test was defined as a score of at least "strong" on the first seven questions of the test, and a total score of 28 points or higher for the rest of the test. The needs assessment of the participants was pivotal in providing important information to formulate the objectives of the workshop and design its content to enhance capacity building in knowledge translation (KT).

After the pre-workshop assessment was analyzed, the following objectives for the workshop were formulated:

1. To enhance the knowledge and skills in EBM of those participants who passed the Fresno test, and to improve the required knowledge and skills of those who did not.

2. To improve the effectiveness of EBHC training beyond critical appraisal.

3. To introduce the concept of KT as an important milestone in teaching EBHC.

4. To highlight the problems of translating evidence into practice to enable participants to address this issue during future EBHC training.

5. To enhance the skills of EBM teachers by building their capacity in knowledge brokering and KT through the creation of communication bridges with policy-makers and administrators, with the goal of overcoming known obstacles to KT.

### The Innovative Teaching Workshop

The workshop program was strategically developed to invest in and consolidate the knowledge and skills of participants who passed the Fresno test. The main strategy was based on having participants teach some of the plenary sessions (Table 1). It also reinforced the basic knowledge and skills of the other participants through modern teaching models such as peer teaching and self-directed learning based on the participants' own scenarios, which they used to formulate answerable questions and search for evidence (Table 1). The workshop was conducted twice. During the second workshop we introduced major changes in the content, teaching methods and methods to evaluate the debate approach and knowledge translation project (KTP) in response to comments from national and international experts in medical education and EBM. The main changes were:

**Table 1:** Innovative Teaching Workshop Content

	Plenary session	Plenary session	Plenary session	Small group/Practical sessions
Day 1	**(30 minutes)** Introduction to the workshop and review of the objectives and contents	**(30 minutes)** Formulating an answerable clinical question as the basis for a successful search strategy	**(30 minutes)** The hierarchy of evidence and the place of RCTs, cohort and case-control studies in decision-making *(Presented by a participant)*	**(2 hours)** Participants presented answerable clinical questions with the PICO format for 20 given scenarios to build a search strategy. The presentations were used to enhance communication skills though critique and comments from the other participants and the facilitator. Participants were introduced to search methods for the Cochrane Library, Medline and other sources of appraised evidence.

Day 2	**(1 hour)** Knowledge translation is the bridge between evidence-based medicine and evidence-based healthcare. Introduction to the knowledge-to-action framework (Review of the KT project)	**(1 hour)** Critical appraisal of RCTs, cohort studies and case-control studies for intervention. Assessment of internal and external validity. Calculation and interpretation of RRR, OR, NNT and NNH	**(45 minutes)** Critical appraisal of diagnostic and prognostic studies and systematic reviews. *(Presented by a participant)* **(30 minutes)** Break-out into groups to debate and review the debate statements	**(1 hour)** Practical session (PICO format) of life scenarios from the participants' clinical practice and hands-on literature search for trials and studies that addressed the PICO question formulated by the participants **(1 hour)** Small group sessions on critical appraisal of different types of trials and studies including the use of an online calculator to calculate NNT, OR and other measures.

Day 3	**(1 hour)** The knowledge-practice gap and the effect of national organizations in reducing the gap (e.g. National Institute for Health and Clinical Excellence)	**(1 hour)** Barriers to and facilitators of knowledge translation	**(2 hours)** First presentation of the knowledge translation project by participants	

Day 4			**(2 hours)** Debates on the first statement by participants	**(2 hours)** Second presentation of the knowledge translation project by participants

Day 5			**(2 hours)** Debates on the second statement by participants	**(30 minutes)** Feedback and post-workshop evaluation **(30 minutes)** Closing remarks

RCT = Randomized controlled trial. PICO = Acronym for **P**opulation, **I**ntervention, **C**omparison, **O**utcome. NNT = Number needed to treat. OR = Odd ratio. RRD = Relative risk reduction. NNH = Number needed to harm. KT = Knowledge translation.

1. The addition of clearly stated objectives for the workshop based on the results of the Fresno test.

2. Modification of the passing score on the Fresno test from an arbitrary score to a score of 85 points.

3. Selection of the topics for the debate statements according to specific criteria as detailed below in the teaching methodology sections for the debate and KTP approached.

4. Assessment of the participants' performance after the debate and KTP components according to pre-stated criteria sent to participants before the workshop began (Additional file 1 and 2).

### Innovative teaching methods

The workshop that served as the focus of this paper consisted of three or four sessions held each day for five days (Table 1). The program included three components, of which the debate and KTP components are described in detail below.

**1. Debate**: The debates were conducted in two sessions of two hours each. The participants in the debate were chosen at random, although all the course participants were informed about the debate topics two weeks before the workshop began. The debate statements were chosen according to the following criteria:

• Relevance to the participants' daily evidence-based practice

• Relation to a common health problem or practice relevant to the participants' community and professional background

• Potential to enable the search for and the appraisal of evidence published in the medical literature, and availability in commonly used databases

• Availability of information about the topic, preferably with level 1 evidence available in the published literature.

The method described by Rubin et al. was used to organize the two debate sessions [4]. In brief, three participants per session formed a group to support the statement and three other participants formed a group to refute it. The participants were given an information sheet describing the debate's objectives, format and the basis for performance assessment (Additional file 1). The whole group for a given debate topic worked together to search for and appraise the evidence, after which each team met to develop its arguments.

At the end of the debate, evaluation was based on the participants' score in four domains: (i) comprehensiveness of their research, (ii) critical appraisal and grading of the evidence used during the debate, (iii) adaptation of evidence to participants' local context, and (iv) the quality of the communication skills used to articulate the evidence to non-medical end users. Participants were evaluated as teams.

Comprehensiveness of the literature search was assessed according to a search strategy conducted and executed by an experienced librarian, and it was further reviewed by the two authors to ensure the clinical relevance of the retrieved articles. The evaluation was based on the percentage of relevant articles of the highest level of evidence available for a given statement that were retrieved by the participants and used during the debate, as compared to the total number of relevant articles retrieved by the librarian and judged to be relevant by the facilitators. In addition, the participants were expected to have retrieved trials or studies relevant to their local community, if available.

For a full score in the critical appraisal domain, the participants were expected to demonstrate that they had examined the internal and external validity of at least one article of the highest level of evidence used in their argument, and to have used high-level evidence to support their argument and refute that of the other team.

For a full score in the third domain, the participants were expected to demonstrate how the evidence retrieved could be adapted to the population in their community, considering cultural and economic background. The participants were also expected to demonstrate their awareness of published studies based on their community or similar communities.

For the fourth domain, the participants were expected to demonstrate their skills in calculating RRR and NNT from one of the highest-level articles and to articulate this information during the debate in a format that was understandable to end-users from a nonmedical background.

The search strategy and the articles retrieved were reviewed and graded by the facilitators before the debates, with a standardized grading system. For each domain the maximum score was 10 points. The two facilitators who assessed performance and assigned the scores had previously received formal training in this field and were the authors of publications on critical appraisal and health technology assessment. The following statements were used for the two debates:

#### Statement 1

"Prescribing antibiotics for upper respiratory tract infection improves patient outcome."

#### Statement 2

"To reduce the incidence of breast cancer in Saudi Arabia, all women above the age of 40 years should undergo mammographic screening."

**Figure 1:**
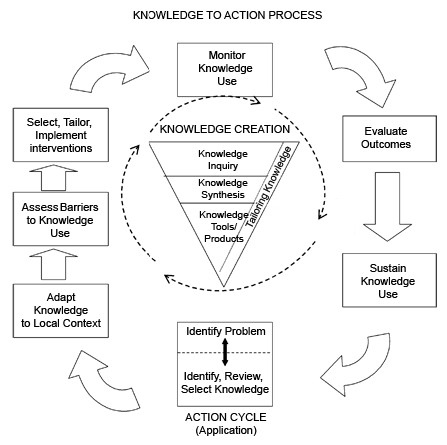
**Knowledge-to-action framework from Graham et al**.

**2. KTP**: Three one-hour lectures were held in which KT, the knowledge-to action framework (Figure 1) [5], the barriers to KT, and the relationship between KT and EBM were defined [6] and the role of national organizations, such as the National Institutes for Health and Clinical Excellence (NICE), in KT were discussed (Table 1). Two weeks before the workshop, the participants were given an information sheet describing the objective, format and the performance assessment for the KTP (Additional file 2), as well as the following clinical statement based on high level of evidence:

"Preconception glycemic control for women with diabetes reduces the incidence of congenital malformations" [7]

The participants were asked to describe how they planned to present the case for establishing a clinical service for preconception care for women with diabetes to the administrator of the health facility where they worked. Four participants from different hospitals, who did not participate in the debate or in peer teaching, presented their cases individually while the rest of the participants acted as hospital administrators and were charged with identifying the logistic difficulties of establishing such a service.

The participants were expected to present their case using the knowledge-to-action framework (Figure 1) [5]. They were also expected to follow the seven steps included in the framework to explain how they planned to execute or communicate each step in their setting, and to detail the involvement of each stakeholder (Additional file 2). At the end of their presentations, the participants were evaluated verbally by the facilitators based on the completeness of their presentations compared to the criteria in Additional file 2.

The topic for the KTP was selected to address important, frequent health problems in the Saudi community: diabetes and congenital abnormalities. Diabetes is becoming a serious public health problem in Saudi Arabia because of the effects of a modern lifestyle and obesity imposed on a background of genetic predisposition [8]. The high prevalence of diabetes increases the burden of congenital abnormalities, which constitute another significant public health problem [9]. Despite the proven effectiveness of preconception glycemic control in significantly reducing the occurrence of congenital abnormalities in the offspring of diabetic mothers [7], the service is provided by only a few health facilities in Saudi Arabia. KT scenarios should be designed to include any part of the knowledge-to-action framework depending on the participants learning needs, provided that it includes the implementation of evidence.

**3. EBM knowledge and skills enhancement**: A series of plenary sessions, small-group discussions and hands-on practical sessions were designed to precede the debates and the KTP in order to ensure that participants had the required knowledge and skills (Table 1). Active contribution by the participants was the main feature of this part of the workshop, which included case scenarios presented by the participants and the delivery of two plenary sessions by two participants in the form of peer teaching (Table 1).

### Workshop Feedback

At the end of the workshop, the participants' opinions about the innovative teaching methods were recorded with a questionnaire based on a 5-point Likert scale from "strongly agree" to "strongly disagree". In addition, they were invited to join a post-workshop discussion group aimed at eliciting their opinions on the debate and the KTP.

## Results

**Figure 2:**
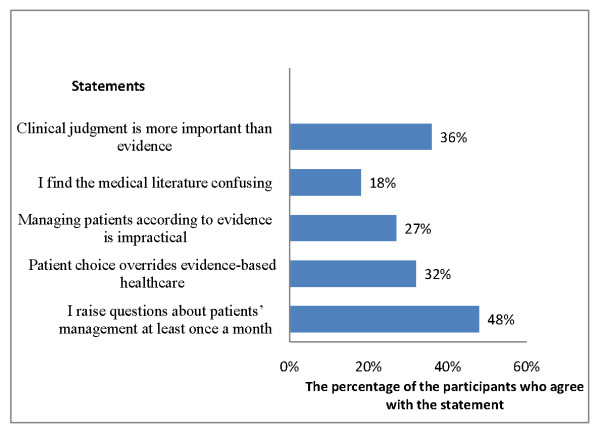
**Participants' practice and attitude towards evidence-based healthcare**.

In the pre-workshop assessment, 13 of the 21 participants passed the Fresno test and all 21 participants were confirmed to have attended at least one workshop on EBM. All participants had a positive attitude towards using EBM in practice; however, they infrequently searched for evidence to answer questions that arose during the management of their patients (Figure 2).

### Debate

The participants were assessed as a group. The mean scores for each of the four domains in the two debate sessions are shown in Table 2.

**Table 2:** Participants score on the debates

Domain	Mean score as a percentage of the full score
Comprehensiveness of literature searching	80%
Appraisal of the evidence used during the debate and examining the internal and external validity of at least one highest level of evidence article.	78%
Clarity of presentation and communication of the main findings to nonmedical end-users	55%
Adaptation of the evidence to the local community by referring to studies or local data and vital statistics	75%

### KTP

Participants had variable success in covering the seven steps of the knowledge-to-action framework, with noticeable difficulties in steps two, three, and four of the action cycle. These steps are related to adapting knowledge to the local context, responding to the barriers and difficulties proposed by the other participants, and tailoring implementation [5]. The scores for clarity of presentation were lower than the scores in the other domains (Table 2). Participants charged with presenting the implementation of the KTP in their health facility faced several difficulties in finding practical solutions to some of the barriers for implementation pointed out by the other participants, such as budgetary constraints, community acceptance of contraception to achieve maternal glycemic control, and the high rate of unplanned pregnancies. Nonetheless, their performance in monitoring and evaluating implementation was satisfactory.

### Workshop Feedback

**Figure 3:**
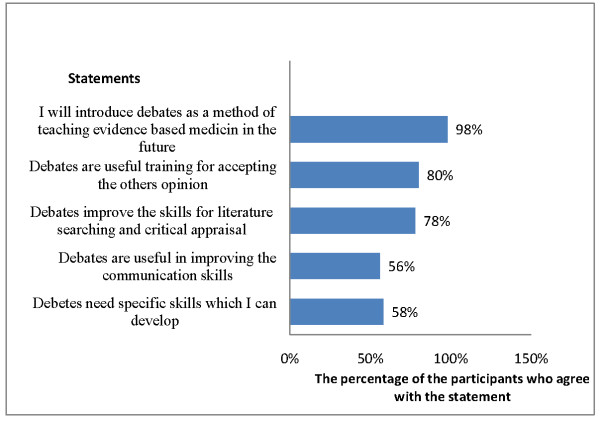
**Participants' opinion about debates as a method of teaching**.

**Figure 4:**
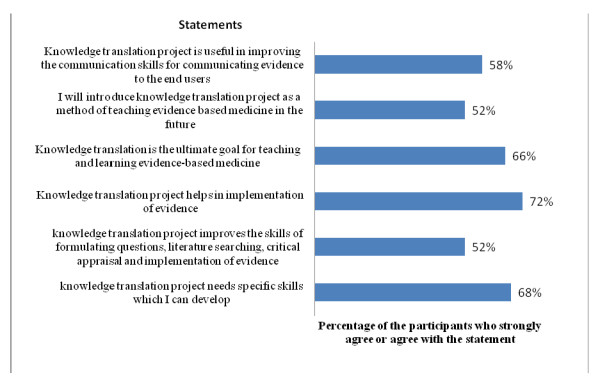
Participants' opinion about the knowledge translation project.

The opinions recorded with the post-workshop questionnaire regarding the two methods of innovative teaching are shown in Figure 3 and 4.

During the post-workshop group discussion, participants voiced the opinion that the debate had excellent potential as a method to enhance their skills in searching, appraising and communicating evidence. They believed that it added a new dimension to their EBM skills by adding purpose to and motivation for the whole exercise. However, they thought that in some instances, advocating a statement that they did not believe compromised their performance. The participants also indicated that their performance would have been better if they had first received a practical demonstration of how to properly conduct the debate.

The participants had a positive opinion about the KTP. They indicated that the project enhanced their understanding of the relationship between evidence and implementation, and broadened their views to include the investigation of public health problems rather than individual patient problems. However, some participants thought that it was outside the scope of their role as doctors to advise the administrator of the health facility about health policy, and felt that efforts to implement evidence should be limited to the individual healthcare provider's practice. They also felt that attempts to extend the implementation of evidence to health policy would be futile because there are no precedents for this type of policy intervention in the Saudi Arabian medical community.

## Discussion

Evidence-based medicine has shifted the paradigm of medical practice by advocating the use of evidence of effectiveness in decision-making for the management of individual patients [10]. However, unlike KT, the concept does not include stakeholders other than the healthcare provider and the patient, nor does it advocate the incorporation of evidence into national health policies [10]. The definition of KT implies the implementation of knowledge to improve not only individual health but also the quality of health services and the healthcare system [11]. The synthesis and dissemination of knowledge to healthcare providers as the sole end-users are not effective in integrating knowledge into healthcare services or the healthcare system, because these processes ignore the other main stakeholders, i.e. policymakers, patients and researchers [12].

The CEBHC-KT was established with a clear vision of how to advance evidence-based healthcare beyond critical appraisal workshops by promoting the concept of KT among the facilitators of EBM and building their skills for knowledge brokering and knowledge transfer [2]. The strategic targeting of the EBM facilitators to promote the concept of KT, capitalizes on their wealth of knowledge and skills in EBM, in addition to their unique position as clinicians and, in some instances, administrators of health facilities throughout the Kingdom. Like capacity building in health research, the concept of capacity building in KT addresses the need to develop and promote the sustainable skills [13] of synthesis, communication and implementation of evidence beyond the individual patient to the community of patients.

The choice of debate as an innovative teaching method was meant to further participants' skills in searching for and appraising evidence. This was achieved by extending the exercise to the use of evidence to support their argument, thus adding purpose and motivation to their previous training. Debates are effective tools for adult learning; hence they are used for teaching purposes in many schools for healthcare professionals [4,14]. They offer a clear goal of winning the debate and give participants control over the learning process. If the topics are well chosen, participants will recognize their relevance to daily practice; in addition, the entertaining nature of the debate enhances the learning experience [4,14].

The statement we chose for the first debate addressed upper respiratory tract infection, a common clinical health problem in which evidence-based decision-making influences individual patient care. The statement for the second debate addressed a public health problem focused on early screening for breast cancer, the most common cancer among Saudi women [15]. These two statements were chosen because they introduced the idea of extending the role of the healthcare provider beyond the clinic to the community and its health problems.

It is difficult to gauge the success of the innovative teaching methods in KT capacity building, because the optimal outcomes of evidence implementation in practice and proof of subsequent changes in the practices and attitudes of the healthcare providers who received this training will take time to materialize. However, the post-workshop feedback provided us with insight into the participants' views about the workshop.

The participants were quite positive about the debates (Figure 3), but their concerns about supporting a statement that they did not believe were raised previously by other learners [4]. Nevertheless, the experience of supporting a statement one does not believe provides participants with endless opportunities for deeper thinking about the position advocated by the statement, and familiarizes them with considering the opinions of others[4]. We believe that the participants' comments about the need for coaching to prepare for the debate are valid, and they will be considered in future workshops.

Although the participants had a positive view of the KTP (Figure 4), they raised genuine concerns about whether stakeholders, including policy-makers, would accept the new role proposed for healthcare providers as knowledge brokers. We believe this concern will persist for a considerable time because it is related to the culture and the role of healthcare providers as seen by the community of healthcare organizations. Managers and policy-makers tend to view the role of care providers as limited to the treatment of patients, with no role in advising health policy based on their knowledge of research evidence or their experience. Nevertheless, interventions such as the ones described in this paper might help policy-makers to change their views regarding this new role for healthcare providers.

## Lessons learned

The main objectives of the innovative teaching methods for KT that we describe here were to provide a recognized training format for KT workshops built on features of adult learning, and to demonstrate the practical opportunities for and barriers to the implementation of evidence in the participants' own environment. The innovative teaching methods were successful in convincing the participants to apply these methods in their own teaching environments (Figures 3 and 4), and it is hoped that their acceptance of these methods will facilitate the spread of a culture of evidence implementation among other stakeholders such as policy-makers. This is imperative in communities that lack national institutions committed to the synthesis of evidence and its formulation in health policies. Our participants recognized that capacity building in KT requires new sets of skills to be built upon their basic knowledge of EBM (Figures 3 and 4), yet despite the challenges, more than half of the participants were willing to learn these skills.

More rigorous measures are needed to evaluate the outcomes of these teaching methods. Potentially useful measures include (i) the number of KTPs implemented in the participants' home practices, (ii) the number of meetings in which clinicians and administrators at different health institutions share relevant evidence and (iii) changes in the attitudes and practices of healthcare providers and other stakeholders in accepting clinicians as knowledge brokers.

We are aware of certain limitation in this study. The limited number of participants who were actively involved in the debates meant that many participants were not able to enjoy and benefit from this activity. This will be addressed by involving more participants in the debate teams in future workshops. Because of time constraints, the number of participants in the KTP was limited. However, the role of the rest of the participants in presenting foreseeable challenges and obstacles to the presenter was, in our view, equally educational and allowed these participants to benefit from the objectives of the KTP.

## Conclusion

Debates and evidence implementation through KTP are generally well accepted by healthcare practitioners as methods by which they can improve their skills in KT.

## Competing interests

The authors declare that they have no competing interests.

## Authors' contributions

HW conceived the idea of the new methods; LA and HW developed the methods further, HW wrote the manuscript. Both authors read and approved the final draft of the manuscript.

## Pre-publication history

The pre-publication history for this paper can be accessed here:

http://www.biomedcentral.com/1472-6920/11/85/prepub

## Supplementary Material

Additional file 1 **Debate objectives and format**. This file describes the format of the debate and how the participants will be assessed. Click here for file

Additional file 2 **Knowledge translation project objectives and format. **This file describes the format of the knowledge translation project and how the participants will be assessed. Click here for file
