# Supplementary material for: Innovative teaching methods for capacity building in knowledge translation
Source: BMC Med Educ. 2011 Oct 14;11:85. doi: 10.1186/1472-6920-11-85 (PMC3215958; doi:10.1186/1472-6920-11-85)
Supplement: Additional file 1 — Debate objectives and format. This file describes the format of the debate and how the participants will be assessed. [file 1472-6920-11-85-S1.DOC]

**Appendix 1**

**Debate objectives and format**:

Dear participant,

The debate is an enjoyable method of learning and it has great relevance to your skills of developing an argument to convince policy makers, administrators and colleagues of adopting an evidence-based approach in their decision and practice. During this workshop you are going to participate in two debates and the following information is for you to prepare for the debates.

The main goal of the debate is to develop an argument for or against an intervention which supports the practice of evidence-based healthcare. The objectives of the debate are:

1. To formulate an answerable question from the given statement (an issue).
2. To conduct a literature search for evidence; for and against the statement.
3. To develop a coherent argument by interpreting the results of the intervention and by making inferences from the magnitude of the outcome using your skills and knowledge of evidence-based medicine.
4. To consider the characteristics of the patients and the local health setting in building the argument.
5. To present your argument in a clear and understandable way to medical professionals as well as to key stakeholders with no medical background

**Format of the debate (the time allowed):**

1. Opening statement from the supporting team (5 minutes).
2. Opening statement from refuting team (5 minutes).
3. First rebuttal from the supporting team (10 minutes)
4. Rebuttal by refuting team (10 minutes).
5. Final rebuttal from supporting team (10 minutes)
6. Final rebuttal from refuting team (10 minutes)
7. Audience questions
8. Voting

The opening statement is a summary of the debate topic and is meant to reflect the general perspective of the team and to stimulate discussion.

**Assessment:**

Your performance during the debate will be assessed according to the following

1. You have to demonstrate an effective search strategy by retrieving at least one article of the highest evidence available on the topic of your debate.
2. You have to demonstrate that you have examined the internal and the external validity of at least one of the highest level of evidence article you retrieved from your search strategy.
3. You have to demonstrate how you will adapt the evidence retrieved to your local community by referring to studies done in your community or similar communities and/or by referring to national data of vital statistics.
4. You should demonstrate your skills in calculating the effect size of the intervention and how you are going to communicate this effect size in terms understandable to end-users from non-medical background.
5. Extra 10 marks will be awarded to the any team who retrieved the more than 50% of the relevant articles to the topic of debate as compared to the number retrieved by our librarian.
