# Supplementary material for: Innovative teaching methods for capacity building in knowledge translation
Source: BMC Med Educ. 2011 Oct 14;11:85. doi: 10.1186/1472-6920-11-85 (PMC3215958; doi:10.1186/1472-6920-11-85)
Supplement: Additional file 2 — Knowledge translation project objectives and format. This file describes the format of the knowledge translation project and how the participants will be assessed. [file 1472-6920-11-85-S2.DOC]

**Appendix 2**

**Knowledge translation project objectives and format**:

Dear participant,

The Knowledge Translation Project (KTP) is a new method of teaching with the main objective of enhancing your skills and knowledge of translating evidence into practice at the level of your community and/or health facility. You are required to develop an argument to convince policy makers, administrators and colleagues of adopting an evidence-based approach related to the statement provided and to make the necessary changes to support the implementation of this evidence. During this workshop you are going to participate in one KTP and the following information is for you to prepare for that.

The main goal of the KTP is to develop an argument for an intervention which supports the practice of evidence-based healthcare. The objectives of the KTP are:

1. To formulate an answerable question from the given statement (an issue).
2. To conduct a literature search for the statement given. Your search should include highest evidence for support of the statement, the anticipated difficulties of implementation of the evidence and the methods for evaluation of implementation.
3. Develop a coherent argument by interpreting the results of the intervention and by making inferences from the magnitude of the outcome using your skills and knowledge of evidence-based medicine.
4. To consider the characteristics of the patients and the local health setting in building the argument.
5. To present your argument in a clear and understandable way to medical professionals as well as to key stakeholders with no medical background

**Assessment:**

Your performance during the KTP will be assessed according to the following

1. You have to demonstrate an effective search strategy by retrieving at least one article of the highest evidence available on the topic of the KTP.
2. You have to demonstrate that you have examined the internal and the external validity of at least one of the highest level of evidence article you retrieved from your search strategy.
3. You have to demonstrate how you will adapt the evidence retrieved to your local community by referring to studies done in your community or similar communities and/or by referring to national data of vital statistics.
4. You should demonstrate your skills in calculating the effect size of the intervention and how you are going to communicate this effect size in terms understandable to end-users from non-medical background.
5. Extra 10 marks will be awarded for including evidence for cost effectiveness of the intervention.
